# Autotetraploid Origin of Chinese Cherry Revealed by Chromosomal Karyotype and In Situ Hybridization of Seedling Progenies

**DOI:** 10.3390/plants12173116

**Published:** 2023-08-30

**Authors:** Yan Wang, Xueou Li, Yan Feng, Juan Wang, Jing Zhang, Zhenshan Liu, Hao Wang, Tao Chen, Wen He, Zhiwei Wu, Yuanxiu Lin, Yunting Zhang, Mengyao Li, Qing Chen, Yong Zhang, Ya Luo, Haoru Tang, Xiaorong Wang

**Affiliations:** 1 College of Horticulture, Sichuan Agricultural University, Chengdu 611130, China; wangyanwxy@sicau.edu.cn (Y.W.); xueouli@sjtu.edu.cn (X.L.); fengyan@stu.sicau.edu.cn (Y.F.); wj798806295@163.com (J.W.); 71281@sicau.edu.cn (J.Z.); l33yona@163.com (Z.L.); wh2sky@163.com (H.W.); hewen0724@gmail.com (W.H.); wuzhiwei@sicau.edu.cn (Z.W.); linyx@sicau.edu.cn (Y.L.); asyunting@sicau.edu.cn (Y.Z.); limy@sicau.edu.cn (M.L.); supnovel@sicau.edu.cn (Q.C.); zhyong@sicau.edu.cn (Y.Z.); luoya945@sicau.edu.cn (Y.L.); htang@sicau.edu.cn (H.T.); 2 Key Laboratory of Agricultural Bioinformatics, Ministry of Education, Chengdu 611130, China; 3 Rural Revitalization Service Center, Agricultural and Rural Bureau of Cuiping District Yibin City, Yibin 644000, China; 4 College of Life Sciences, Sichuan Agricultural University, Ya’an 625014, China; chentao296@163.com

**Keywords:** Chinese cherry, *Cerasus pseudocerasus* (Lindl.) G.Don, autotetraploid origin, chromosomal karyotype, rDNA-FISH, GISH

## Abstract

Polyploidy is considered a driving force in plant evolution and diversification. Chinese cherry [*Cerasus pseudocerasus* (Lindl.) G.Don], an economically important fruit crop native to China, has evolved at the tetraploid level, with a few pentaploid and hexaploid populations. However, its auto- or allo-polyploid origin remains unclear. To address this issue, we analyzed the ploidy levels and rDNA chromosomal distribution in self- and open-pollinated seedling progenies of tetraploid and hexaploid Chinese cherry. Genomic in situ hybridization (GISH) analysis was conducted to reveal the genomic relationships between Chinese cherry and diploid relatives from the genus *Cerasus*. Both self- and open-pollinated progenies of tetraploid Chinese cherry exhibited tetraploids, pentaploids, and hexaploids, with tetraploids being the most predominant. In the seedling progenies of hexaploid Chinese cherry, the majority of hexaploids and a few pentaploids were observed. A small number of aneuploids were also observed in the seedling progenies. Chromosome 1, characterized by distinct length characteristics, could be considered the representative chromosome of Chinese cherry. The basic Chinese cherry genome carried two 5S rDNA signals with similar intensity, and polyploids had the expected multiples of this copy number. The 5S rDNA sites were located at the per-centromeric regions of the short arm on chromosomes 4 and 5. Three 45S rDNA sites were detected on chr. 3, 4 and 7 in the haploid complement of Chinese cherry. Tetraploids exhibited 12 signals, while pentaploids and hexaploids showed fewer numbers than expected multiples. Based on the GISH signals, Chinese cherry demonstrated relatively close relationships with *C. campanulata* and *C. conradinae*, while being distantly related to another fruiting cherry, *C. avium*. In combination with the above results, our findings suggested that Chinese cherry likely originated from autotetraploidy.

## 1. Introduction

Polyploidy plays a significant role in the evolution of eukaryotes, resulting in organisms with more than two complete sets of chromosomes [1]. In plant organisms, processes such as mutation, recombination, selection, and isolation contribute to species evolution, particularly in flowering plants [2]. Polyploidy provides opportunities for chromosomal rearrangements, which aid in individual adaptation [3,4]. The incidence of polyploidy has been widely estimated in the literature, with reported ranges varying from 30% to 35% up to 70% for angiosperms [5]. Stebbins recognized four types of polyploids based on genetic and cytogenetic criteria: autopolyploids, segmental allopolyploids, true or genomic polyploids, and autoallopolyploids; and the first three of these were considered major types [6]. Ramsey and Schemske indicated that the rate of autopolyploidy formation may often be higher than the rate of allopolyploid formation [7]. Generally, autopolyploids have chromosome sets originating from the same species, while allopolyploids are formed through interspecific hybridization, leading to multiple genome origins [8]. Homogenetic association and multivalents during meiosis are characteristic of autopolyploids, whereas allopolyploids exhibit restricted segregation and predominant chromosome pairing between the same original genome [6].

The genus *Cerasus* (Rosaceae) is mainly distributed in the north temperate zone, with China having the largest number of species in this genus [9]. Chinese cherry [*Cerasus pseudocerasus* (Lindl.) G.Don] is an economically important cherry species within the *Cerasus* genus, along with sweet cherry (*C. avium*), sour cherry *(C. vulgaris*) and nanking cherry (*C. tomentosa*) [10,11]. Sweet cherry and nanking cherry are diploids (2*n* = 2*x* = 16), while sour cherry is an allotetraploid (2*n* = 4*x* = 32) [12], deriving from natural interspecific hybridization between *C. avium* and *C. fruticosa* [13]. Based on our previous reports, Chinese cherry populations have a stable ploidy level with tetraploid (2*n* = 4*x* = 32), although pentaploids (2*n* = 5*x* = 40) and hexaploids (2*n* = 6*x* = 48) also exist in nature [14,15]. Despite karyotype analysis of Chinese cherry [14], the origin of its genome remains a mystery, whether it resulted from genome duplication or interspecific hybridization.

One approach to determine the genome composition of polyploids is to investigate chromosome pairing behavior during meiosis. Autopolyploids exhibited multivalent through homologous chromosome pairing, leading to gametes with varying numbers of chromosomes [16]. Karyotype analysis aids in understanding chromosome morphology and homologous pairing, providing another means to study the auto- or allo-polyploidy origin of a species. In species with small and identical chromosomes, physical mapping of ribosomal DNA (rDNA) loci facilitates homologous pairing. The fluorescence in situ hybridization (FISH) technique, employing repetitive DNA probes, has been widely used to identify auto- or allopolyploids in plants [17,18,19]. Genomic in situ hybridization (GISH), a variant of FISH, utilizes whole genomic probes for suppressive hybridizations to visualize individual genomes in hybrids and polyploids, illustrating the genetic relationship between relative species [20,21]. The hybridization signals not only represent common DNA sequences, but also demonstrate genomic homology between the two species [22].

In this study, we conducted a statistical analysis of the ploidy level and chromosome number of Chinese cherry seedlings, including self- and open-pollinated progenies. We then employed rDNA repetitive sequences as probes to identify homologous chromosomes and establish cytogenetic markers for karyotyping analysis. GISH analysis was also performed to investigate the degree of genomic homology between the Chinese cherry and its diploid relative species. This work aims to provide strong evidence supporting the auto- or allo-tetraploid origin of Chinese cherry.

## 2. Results

### 2.1. Ploidy Levels in Seedling Progenies of Polyploid Chinese Cherry

We confirmed the ploidy levels of parental trees of Chinese cherry accessions by using root tips, young leaves, and flower buds (Table 1). Six Chinese cherry accessions, including XC1, BJ7, NY1, TH2, BZ, and LY4, were all tetraploid with chromosome numbers 2*n* = 4*x* = 32. Conversely, PD3 demonstrated a hexaploid condition with 2*n* = 6*x* = 48 (Figure S1). Subsequently, we obtained 54 seeds from nine self-pollinated and 53 seeds from two open-pollinated progenies of Chinese cherry, respectively. The ploidy level and chromosome number of metaphase cells were then determined using the root tips of germinated seeds.

**Figure 1:**
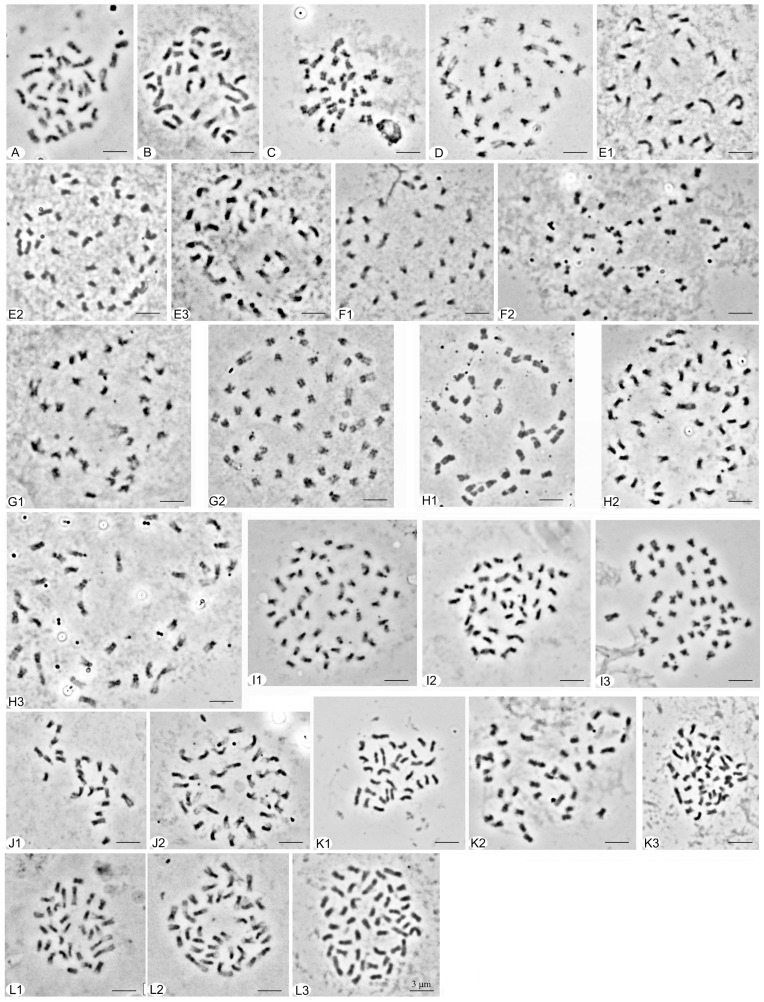
Chromosome numbers (2*n*) of self- and open-pollinated progenies in polyploid Chinese cherry. (**A**–**D**) 4*x* = 32, (**A**) Miyi 3; (**B**) Xichang 2; (**C**) Mengzi 3; (**D**) Anqiu 3. (**E**) Xichang 1 (E1: 4*x* = 32; E2: 5*x* = 40; E3: 4*x* + 1 = 33); (**F**) Bijie 7 (F1: 4*x* = 32; F2: 5*x* = 40); (**G**) Taihe 2 (G1: 4*x* = 32; G2: 6*x* − 1 = 47). (**H**) Nayong 1 (H1: 4*x* = 32; H2: 6*x* = 48; H3: 4*x* + 1 = 33); (**I**) Puding 3 (I1: 6*x* = 48; I2: 5*x* = 40; I3: 6*x* + 1 = 49); (**J**) Nanjing 2 (flowering cherry) (J1: 3*x* = 24, J2: 4*x* = 32); (**K**) Bazhong (K1: 4*x* = 32; K2: 5*x* = 40; K3: 6*x* = 48); (**L**) Luoyang 4 (L1: 4*x* = 32; L2: 5*x* = 40; L3: 6*x* = 48). Scale bar represents 3 μm.

In terms of the self-pollinated progenies of tetraploid Chinese cherry, the chromosome number in 131 metaphase cells of 34 seeds was found to be 2*n* = 4*x* = 32, which accounted for 85% (Table 1, Figure 1A–H). Two cells from two seeds exhibited a pentaploid condition with a chromosome number of 5*x* = 40, representing 5% of the total. In addition, one seed displayed a hexaploid state (2*n* = 6*x* = 48) in 13 cells. Aneuploid was also observed, with 33 chromosomes (2*n* = 4*x* + 1) (Figure 1E3,H3) and 47 chromosomes (2*n* = 6*x* − 1) (Figure 1G2), in five cells from three seeds. These aneuploid conditions accounted for 7.5% of the progenies.

Regarding the 14 seeds obtained from self-pollinated progenies of hexaploid Chinese cherry, 80 euploid cells were identified in 13 seeds, while one seed exhibited four aneuploid cells (Table 1, Figure 1I1–I3). Among the nine seeds, 65 cells exhibited a hexaploid state with a chromosome number of 2*n* = 6*x* = 48, representing 64.29% of the total. Additionally, 15 cells from four seeds displayed a pentaploid state with 40 chromosomes, accounting for 28.57%. Furthermore, four cells in one seed presented a chromosome number of 49 (2*n* = 6*x* + 1).

No aneuploid was detected among 53 seeds from open-pollinated progenies of tetraploid Chinese cherry (Table 1, Figure 1K,L). Out of the total of 176 metaphase cells examined, 39 seeds exhibited a tetraploid condition, representing 75.38%. Pentaploid and hexaploid states were observed in 10 seeds (44 cells) and two seeds (seven cells), respectively, accounting for 18.87% and 3.77% of the progenies.

### 2.2. Karyotype Characteristics of Polyploid Chinese Cherry

**Figure 2:**
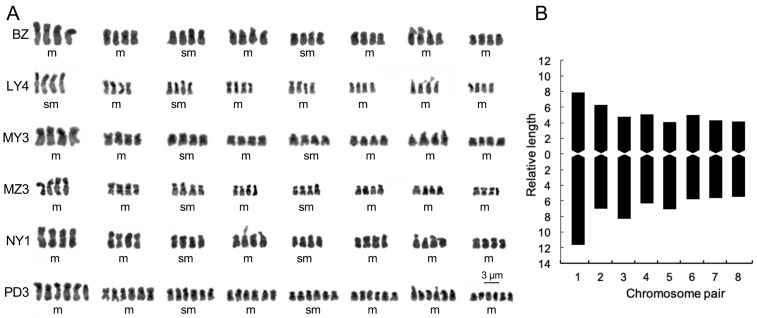
Chromosomal karyotype (**A**) and idiogram (**B**) in polyploid Chinese cherry. m: metacentric, sm: submetacentric.

A total of 77 metaphase cells with clear chromosome morphology were selected for karyotypic analysis (Table 2, Figure 2). The karyotype formula for tetraploid was 2*n* = 4*x* = 32 = 24 m + 8 sm, and for hexaploid it was 2*n* = 6*x* = 48 = 42 m + 6 sm. In tetraploid cells, there were four or eight satellites on the short arms of chromosomes 4 and 7, while in hexaploid cells, there were six satellites on the short arms of chromosome 7. The relative length composition in tetraploid cells mainly consisted of 4L + 8M2 + 16M1 + 4S, with a few cases having 4L + 8M2 + 20M1. In hexaploid cells, the relative length constitution was 6L + 12M2 + 30M1 for hexaploid. Chromosome 1 had the largest relative length, which was 47.14% to 69.21% larger than chromosome 2 in tetraploid cells, and 39.15% larger in hexaploid cells (Table S1). The index of the karyotypic asymmetry ranged from 58.55% to 65.82% in tetraploid cells and 56.98% in hexaploid cells. LY4 has the highest ratio of longest to shortest chromosome (2.39), followed by NY1 (2.32), while BZ had the smallest value (2.01). The mean arm ratio (MAR) varied from 1.33 to 1.46 in polyploid Chinese cherry. Both tetraploid and hexaploid Chinese cherries had a “1B” karyotype type.

### 2.3. rDNA Chromosomal Distribution in Polyploid Chinese Cherry

**Figure 3:**
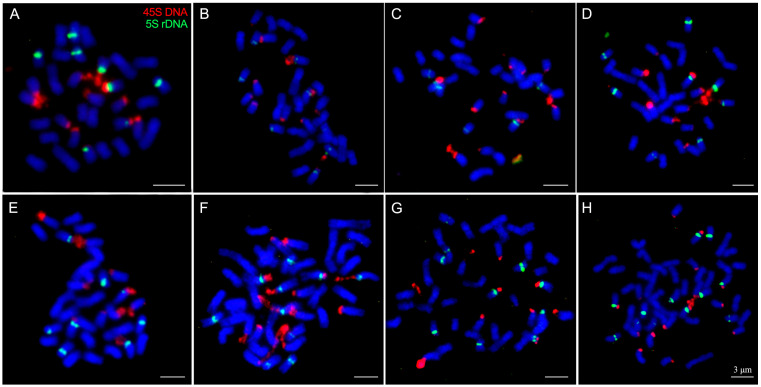
rDNA-FISH on metaphase chromosomes in polyploid Chinese cherry. (**A**) Xichang 1 (4*x*), (**B**) Mengzi 3 (4*x*), (**C**) Bijie 7 (4*x*), (**D**) Luoyang 4 (4*x*), (**E**) Bazhong (4*x*), (**F**) Bazhong (5*x*), (**G**) Puding 3 (5*x*), (**H**) Puding 3 (6*x*). The red and green signals indicate 45S and 5S rDNA sites, respectively. Scale bar represents 3 μm.

**Figure 4:**
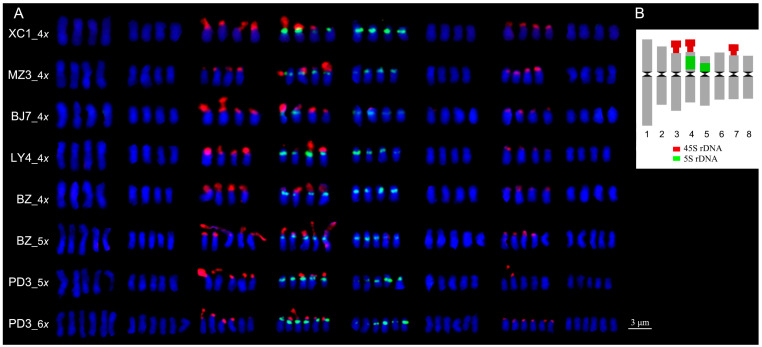
Karyotype (**A**) and idiogram in haploid complement (**B**) based on rDNA-FISH distribution in Chinese cherry.

The fluorescence in situ hybridization (FISH) technique was employed to map the 5S and 45S rDNA on chromosomes, as illustrated in Figure 3 and Figure 4. The results revealed that 5S rDNA sites were located at the proximal centromere of the short arms on chromosomes 4 and 5 in Chinese cherry. In the haploid complement, two loci with similar intensity were observed (Figure 3 and Figure S2). Generally, the tetraploid Chinese cherry carried 8 sites with similar intensities, except for BJ7, which had 6 sites (Figure 3A–E). The numbers of 5S rDNA sites in the pentaploid and hexaploid were 10 and 12, respectively (Figure 3F–H).

The 45S rDNA was found to be localized at the secondary constrictions of short arms on chromosomes 3, 4 and 7, associated with the satellites. The triploid flowering cherry and tetraploid Chinese cherry displayed 9 (Figure S3) and 12 signals (Figure 3A–E), respectively. In pentaploid and hexaploid Chinese cherry, a relatively high level of polymorphism was identified concerning the number of 45S rDNA sites. For instance, pentaploid BZ had 15 45S rDNA signals, while PD3 exhibited 10 signals (Figure 3F,G). The hexaploid PD3 carried 16 signals (Figure 3H), which is less than the expected 18 signals with the increasing ploidy level. Additionally, the intensity of 45S rDNA signals varied across different chromosomes, unlike 5S rDNA. It is evident that the 45S rDNA sites in chr. 3 and 4 were larger and stronger than those in chr. 7 (Figure 4A). Based on rDNA chromosomal distribution, a preliminary karyotype idiogram of the haploid complement of Chinese cherry has been established, as depicted in Figure 4B. One out of the two 5S rDNA sites was co-localized with a 45S rDNA signal on the short arm of chromosome 4. Therefore, four pairs of chromosomes in Chinese cherry can be clearly identified based on rDNA loci.

### 2.4. GISH Analysis between Chinese Cherry and Cerasus Diploid Relatives

**Figure 5:**
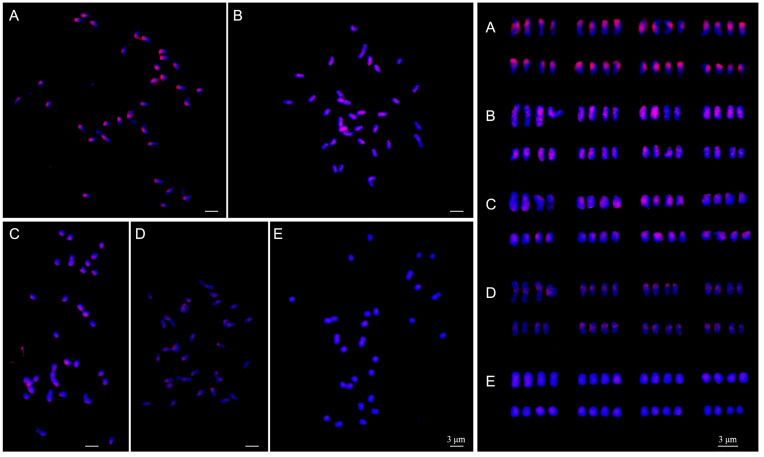
GISH (**A**–**E** **left**) and karyotypes (**A**–**E** **right**) on metaphase chromosomes in Chinese cherry. Genomic probes were from *C. pseudocerasus* (**A**), *C. campanulata* (**B**), *C. conradinae* (**C**), *C. serrulata* var. *lannesiana* (**D**), and *C. avium* (**E**), respectively. Based on the traditional classification [9], *C. pseudocerasus* is assigned into section *Lobopetalum* subgenus *Cerasus*, *C. campanulata* and *C. conradinae* into sect. *Serrula*, *C. serrulata* var. *lannesiana* into sect. *Sargentiella*, and *C. avium* into sect. *Cerasus* subgenus *Cerasus*, respectively. Scale bar represents 3 μm.

Genomic in situ hybridization (GISH) analysis was conducted to determine the genomic homology between the Chinese cherry and its diploid relatives in the genus *Cerasus*. The genomic probe derived from the Chinese cherry exhibited the most pronounced signals, which were observed primarily in the centromeric regions and sparsely distributed along the short arms of 32 chromosomes (Figure 5A). The hybridization signals generated by both *C. campanulata* and *C. conradinae* probes were detected in the centromeric to the subterminal region of the short arms across all chromosomes, with signal intensities weaker than those observed with the Chinese cherry probe (Figure 5B,C). Tol- or proximal centromeric regions displayed moderate signals when probed with *C. serrulata* var. *lannesiana* (Figure 5D). Conversely, the hybridization signals obtained with the *C. avium* probe were the weakest among the tested cherry species on the chromosomes of Chinese cherry (Figure 5E).

## 3. Discussion

Polyploidy has long been considered a ubiquitous phenomenon driving plant evolution and diversification [1]. Our previous reports have confirmed the polyploid origin of Chinese cherry [14], but further clarification regarding its auto- or allo-polyploidy origin is required. In this study, we analyzed the ploidy level and chromosome number of self- and open-pollinated progenies of tetraploid and hexaploid Chinese cherry. Among the progenies of tetraploid Chinese cherry, tetraploids, pentaploids, and hexaploids accounted for 78.49%, 12.90%, and 3.23%, respectively. In terms of the progenies of hexaploid Chinese cherry, 64.29% were hexaploids and 28.57% were pentaploids. During meiosis, autopolyploids usually exhibit multivalents formation, or tetrasomic ratios [16], while allopolyploids mainly form bivalents [23,24]. Our results suggested that meiotic chromosome pairing in tetraploid Chinese cherry was likely characterized by bivalent plus bivalent, and trivalent plus univalent configurations. For hexaploid Chinese cherry, meiotic pairing involved trivalent plus trivalent, and bivalent plus quadrivalent configurations. Therefore, tetraploids accounted for the largest proportion among the progenies of tetraploid Chinese cherry, followed by a few pentaploids and hexaploids. Similarly, the proportion of hexaploids was much higher than pentaploids among the progenies of hexaploid Chinese cherry. Based on the above information, it is likely that Chinese cherry originated from autopolyploidy rather than allopolyploidy.

Due to the small size (i.e., 1.54~5.03 μm for Chinese cherry) and morphological similarity of cherry chromosomes, chromosome-specific markers such as rRNA genes (Plant rDNA database, www.plantrdnadatabase.com, accessed on 5 June 2023) provided by FISH are needed for the identification of individual chromosomes [25]. This identification is critical for tracking homo- or homoeologous chromosomes among species and polyploids. In addition, the number and size polymorphism of rDNA signals in plants can provide alternative possibilities of auto- or allopolyploid origin [26]. For example, *Hepatica nobilis* var. *pubescens* is suggested as an autotetraploid derivative of the diploid *H. nobilis* var. *japonica*, which is supported by the high degree of similarity of the four homologues of all chromosome pairs, including pairs 6 and 7 that carry rDNA [27]. The size polymorphism of 5S rDNA signals in *Fragaria corymbosa* possibly supports the hypothesis of allotetraploid origin [26]. In the present study, two 5S rDNA signals were observed in the haploid complement of Chinese cherry, being consistent with previous reports in *Prunus* [28,29] and *Rosa* [30] species. However, this number differs from most genera in the Rosaceae family, such as *Fragaria* [26,31], *Rubus* [32], and *Pyrus* [33], which typically have a constant number of one cluster per haploid set. In polyploid Chinese cherry, the numbers of 5S rDNA signals increased proportionally with the increase in whole genome copy number. Furthermore, the 5S rDNA signals exhibited similar intensities in the haploid chromosome set of Chinese cherry. Thus, the results based on 5S rDNA-FISH further support the autopolyploid origin of Chinese cherry.

Compared with 5S rDNA sites, 45S rDNA sites appear to be highly variable in numbers and intensities in Rosaceae plants, such as *Fragaria* [26,31], *Rubus* [34], and *Sanguisorba* [35]. Double-color FISH of 45S/5S rDNA allowed the distinguishing of three different chromosome groups within two cherry rootstocks [28]. Here, three 45S rDNA signals were detected in the haploid chromosome set of flowering cherry and Chinese cherry, which is consistent with the findings in *Prunus* plants, including cherry rootstocks [28], peach [36], and almond [37]. All tetraploids displayed the expected multiples of this copy number, whereas pentaploids and hexaploids exhibited fewer numbers than expected. This might indicate that some signals might be too weak to detect in higher-ploidy Chinese cherries. Lavia [17] found that the number of 45S rDNA loci in triploids varied between individuals, even different cells of the same individual. In the population of *Lippia alba*, the signals of tetraploids and hexaploids were fewer than expected compared with diploids [19]. Generally, 45S rDNA sites are located at the terminal regions of the short arms, like with satellites. These regions are prone to chromosome structural variation and are affected by gene dosage. Consequently, there is a trend towards a reduction in the number of 45S rDNA sites per haploid complement in polyploids [38]. Our findings in higher polyploid Chinese cherries were consistent with this point of view.

In plants, the 45S rDNA genes are located within the nucleolar organization regions (NORs), while the 5S rRNAs are localized outside the NORs. Generally, 5S and 45S rDNA sites tended to be distributed independently on chromosomes because their functional divergences would require different physical locations [39]. In this study, rDNA-FISH signals accurately identified chromosome pairs 3, 4, 5, and 7 in Chinese cherry. In terms of chromosome position, 5S rDNA sites are in proximal regions while 45S rDNA repeats are localized at (sub)-terminal regions on short arms, which is highly conserved in the Rosaceae species including *Cerasus* [25,38,40]. Interestingly, there is one 5S and one 45S rDNA site co-localized on short arms of chromosome 4 in a haploid set of Chinese cherry. The co-localization of 5S and 45S rDNA sites has also been reported in related genera such as *Prunus subhirtella* [28], *Fragaria* [26,31,41], and *Rosa* [42,43,44]. The closely linked rDNAs were regarded as an ancestral condition, and the positional changes of the rDNA linkage might have been caused by chromosome arrangements or transposition [45]. Additionally, chromosome pair 1, which exhibited the largest relative length, differed from the other seven pairs of chromosomes and could be considered the representative chromosome of Chinese cherry, as described by Salesses [46]. Similar findings have also been reported in other drupe fruits [15]. Thus, the combined karyotype and FISH results successfully identified five pairs of chromosomes in Chinese cherry. With the rapid development of whole-genome sequencing, it is now possible to computationally identify oligos specific to a repetitive DNA sequence, to a specific chromosomal region, or to an entire chromosome [47]. In the future, the oligo-FISH technique could be utilized for the accurate identification of each chromosome in Chinese cherry.

The GISH technique has been successfully used to illustrate the relationships between related species in woody plants with small chromosomes [20,21]. The tetraploid Chinese cherry genome size was estimated to be 294 Mb, with repetitive sequences accounting for about 31.95% of the genome [48]. The self-GISH signals on Chinese cherry chromosomes were mainly located at the proximal centromeric or terminal regions on the short arms, exhibiting the strongest intensities. Similar hybridization patterns, probed by *C. campanulata* and *C. conradinae* from section *Serrula* of subgenus *Cerasus*, suggested that they shared more common DNA sequences with Chinese cherry than the other species examined. Phylogenomics studies have suggested that the cultivated fruiting cherry species have independent origins, especially between sweet cherry and Chinese cherry [49,50]. In this study, *C. avium* showed a relatively distant genetic relationship with Chinese cherry, as revealed by GISH signals, supporting our previous results.

## 4. Materials and Methods

### 4.1. Plant Materials and Chromosome Preparation

Seeds from self-pollinated progenies of nine Chinese cherry landraces, including Miyi 3, Xichang 1, Xichang 2, Mengzi 3, Anqiu 3, Bijie 7, Nayong 1, Taihe 2, and Puding 3, were collected for this study. Seeds from open-pollinated progenies of Chinese cherry (Bazhong, Luoyang 4) were also included (Table 1). A flowering cherry (Nanjing 2) was utilized for the identification of ploidy level and FISH analysis. These trees have been planted at the Cherry Germplasm Repository of Sichuan Province (Sichuan Agricultural University), Chengdu, China, for over eight years.

Root tips were obtained from germinated seeds or softwood cuttings at 25 °C and pretreated with 2 mol·L^−1^ 8-hydroxyquinoline at 4 °C for 4 h. Then the root tips were fixed in Carnoy’s I solution (absolute ethanol: glacial acetic acid = 3:1, *v*/*v*) at 4 °C for 24 h. The fixed root tips were treated with an enzymatic mixture consisting of 4% (*w*/*v*) Cellulase RS and 2% (*w*/*v*) Pectinase Y23 at 37 °C for 1 to 2 h. Afterward, they were washed with 70% ethanol, followed by dropping the cell suspension onto slides using pure acetic acid at a volume of 7 to 10 μL per slide. Young leaves and flower buds were only treated with fixed liquid, and the subsequent steps were identical to the aforementioned process. Each seed was treated as an individual, and the cells in mitotic metaphase were captured using an Olympus BX53 microscope (Olympus Optical Co., Ltd., Tokyo, Japan) equipped with a DP-70 CCD camera. The images obtained were processed using Cellsens Standard software (Version 1.2.1) for subsequent analysis.

### 4.2. Probe Labeling and Fluorescence In Situ Hybridization

Total genomic DNA was extracted using a modified CTAB protocol [51]. 45S rDNA was amplified using primers 45S-F1 (5′-TAC CTG GTT GAT CCT GCC AGT A-3′) and 45S-R1 (5′-CAA TGA TCC TTC CGC AGG TTC A-3′) [52] with template DNA from Chinese cherry and labeled with biotin-16-dUTP nick translation (Roche Applied Science, Indianapolis, IN, USA). 5S rDNA probe was labeled using digoxigenin probe synthesis kit (Roche Applied Science, Indianapolis, IN, USA) with primers 5S1 (5′-GGA TGC GAT CAT ACC AG CAC-3′) and 5S2 (5′-GGG AAT GCA ACA CGA GGA CT-3′) [53]. The genomic DNA probes of Chinese cherry and four *Cerasus* diploid relatives were labeled by nick translation with biotin-16-dUTP (Roche Applied Science, Indianapolis, IN, USA).

FISH and GISH procedures were performed according to the method described by Wang et al. [40]. Prior to hybridization, the slides were treated with 100 μg·mL^−1^ RNase A solution (Sigma-Aldrich, St Louis, MO, USA) at 37 °C for 1 h and 1 mg·mL^−1^ Proteinase K (Sangon, Shanghai, China) at 37 °C for 30 min. Subsequently, the slides were denatured in 70% formamide at 70 °C for 2 min and dehydrated in an ice-cold ethanol series of 70%, 95%, and 100% for 5 min each.

The hybridization mixture consisted of labeled probes, deionized formamide, dextran sulfate, 2× SSC, and sodium dodecyl sulfate. The mixture was denatured at 100 °C for 10 min, followed by immediate ice bathing for 10 min. Then, 30 μL of the mixture was applied to each slide and incubated at 37 °C for 20 h in a humid chamber. Afterward, the slides were post-hybridization washed twice in 2× SSC for 5 min each. Anti-DIG fluorescein isothiocyanate (FITC, Roche) and tetramethyl-rhodamine isothiocyanate (TRITC, Thermo Fisher Scientific Inc, Darmstadt, Germany) were used for signal detection. The slides were washed by 2× SSC and ddH_2_O once. The chromosome slides were counterstained with 2 ng·μL^−1^ 4′,6-diamidino-2-phenylindole (DAPI, Solarbio, Beijing, China) in Vectashield antifade (Solarbio, Beijing, China) medium for 10 min. The images were captured with a DP-70 CCD camera mounted on a fluorescence microscope (Olympus BX53, Tokyo, Japan) and processed by Cellsens Standard Software (Version 1.2.1).

### 4.3. Karyotype Analysis

At least three metaphase cells with clear chromosome morphology were selected for karyotype analysis. The position of the centromere was determined, and the chromosome karyotype was analyzed based on the size and morphological characteristics. Relative chromosome length, arm ratio and chromosome type were calculated using Levan’s nomenclature [54]. The karyotype asymmetry coefficients were calculated, and the karyotypes were classified according to Stebbins’ criteria [55].

## 5. Conclusions

In summary, tetraploid, pentaploid, and hexaploid ploidy levels were observed in both self- and open-pollinated progenies of tetraploid Chinese cherry, with tetraploids being the most abundant. Hexaploid and pentaploid ploidy levels were found in the progenies of hexaploid Chinese cherry. Chromosome 1, characterized by distinct relative length characteristics, is distinguishable from the other seven pairs. In the haploid chromosome set of Chinese cherry, two 5S and three 45S rDNA sites were detected, enabling accurate identification of chromosomes 3, 4, 5 and 7. Genomic in situ hybridization analysis revealed genomic homology between the Chinese cherry and its diploid relatives from the genus *Cerasus*. Based on the combined evidence of ploidy level, karyotype analysis, and rDNA distribution pattern, we conclude that Chinese cherry is likely an autotetraploid.

## Figures and Tables

**Table 1:** Chromosome numbers (2*n*) of self- and open-pollinated progenies of polyploid Chinese cherry.

Code	Locality	Number		Parental Trees (2*n*)	Self- and Open-Pollinated Progeny Seeds (2*n*)					
		Bagged Flowers	Seeds		4*x* = 32	4*x* + 1 = 33	5*x* = 40	6*x* − 1 = 47	6*x* = 48	6*x* + 1 = 49
Self-pollinated										
MY3	Miyi, Sichuan	14	32	ND	22/5	-	-	-	-	-
XC1	Xichang, Sichuan	14	NA	4*x* = 32	8/3	1/1	1/1	-	-	-
XC2		16	36	ND	15/11	-	-	-	-	-
MZ3	Mengzi, Yunnan	16	36	ND	23/3	-	-	-	-	-
AQ3	Anqiu, Shandong	11	9	ND	35/3	-	-	-	-	-
BJ7	Bijie, Guizhou	29	16	4*x* = 32	3/2	-	1/1	-	-	-
TH2	Taihe, Anhui	26	13	4*x* = 32	1/1	-	-	1/1	-	-
NY1	Nayong, Yunnan	17	40	4*x* = 32	24/6	3/1	-	-	13/1	-
PD3	Puding, Yunnan	13	25	6*x* = 48	-	-	15/4		65/9	4/1
Open-pollinated										
BZ *	Bazhong, Sichuan	NA	NA	4*x* = 32	164/32	-	40/6	-	6/1	-
LY4	Luoyang, He’nan	NA	NA	4*x* = 32	12/7	-	4/4	-	1/1	-

Note: The numbers (n/n) indicate the number of metaphase cells/number of progeny seeds. -: not available. ND: not detected. * represents a wild Chinese cherry accession.

**Table 2:** Comparison of karyotypic characters of representative polyploid Chinese cherry accessions.

Code	Karyotype Formula	Relative Length Constitution	As.K (%)	Lc/Sc	MAR	Type
MY3	2*n* = 4*x* = 32 = 24 m (4SAT) + 8 sm	4L + 8M2 + 16M1 + 4S	65.82	2.16	1.43	1B
MZ3	2*n* = 4*x* = 32 = 24 m + 8sm	4L + 8M2 + 20M1	58.56	2.10	1.43	1B
NY1	2*n* = 4*x* = 32 = 24 m (8SAT) + 8 sm	4L + 8M2 + 16M1 + 4S	59.04	2.32	1.46	1B
BZ	2*n* = 4*x* = 32 = 24 m (8SAT) + 8 sm	4L + 8M2 + 20M1	57.82	2.01	1.38	1B
LY4	2*n* = 4*x* = 32 = 24 m (4SAT) + 8 sm	4L + 8M2 + 16M1 + 4S	58.55	2.39	1.40	1B
PD3	2*n* = 6*x* = 48 = 42 m (6SAT) + 6 sm	6L + 12M2 + 30M1	56.98	2.03	1.33	1B

Note: The relative length constitution was determined by the index of relative length (IRL). L: IRL ≥ 1.25; M2: 1.01 ≤ IRL < 1.25; M1: 0.76 ≤ IRL < 1.01; S: 0.67 ≤ IRL < 0.76. The IRL values for each chromosome were shown in Table S1. As.K (%) = index of the karyotypic asymmetry; Lc/Sc = longest chromosome/shortest chromosome; MAR: mean arm ratio (arm ratio = length of the long arm/length of the short arm). m: metacentric; sm: submetacentric.

## Data Availability

Not applicable.

## Supplementary Materials

The following supporting information can be downloaded at: https://www.mdpi.com/article/10.3390/plants12173116/s1, Table S1: Chromosome parameters in polyploid Chinese cherry. Figure S1: Chromosome number (2*n*) of parental trees in Chinese cherry. A: Xichang 1 (4*x* = 32), B: Bjjie 7 (4*x* = 32), C: Bazhong (4*x* = 32), D: Taihe 2 (4*x* = 32), E: Nayong 1 (4*x* = 32), F: Puding 3 (6*x* = 48), G: Luoyang 4 (4*x* = 32). Scale bar represents 3 μm. Figure S2: 5S rDNA-FISH on metaphase chromosomes in polyploid Chinese cherry. Green signals indicate 5S rDNA sites. Scale bar represents 3 μm. Figure S3: 45S rDNA-FISH on metaphase chromosomes in a triploid flowering cherry (Nanjing 2). Red arrows indicate 45S rDNA sites. Scale bar represents 3 μm.
