# Supplementary material for: Autotetraploid Origin of Chinese Cherry Revealed by Chromosomal Karyotype and In Situ Hybridization of Seedling Progenies
Source: Plants (Basel). 2023 Aug 30;12(17):3116. doi: 10.3390/plants12173116 (PMC10490022; doi:10.3390/plants12173116)
Supplement: Supplementary file 1 [file plants-12-03116-s001.zip › plants-2517973-supplementary.pdf]

Table S1. Chromosome parameters in polyploid Chinese cherry.

Code	Chr. no.	Relative length (%)	Index of relative length	Arm ratio	Centromere index	Type	Code	Chr. no.	Relative length (%)	Index of relative length	Arm ratio	Centromere index	Type
MY3	1	7.80+12.03=19.82	1.59	1.54	39.33	m	LY4	1	7.95+14.46=22.42	1.69	1.82	35.48	sm
	2	6.38+7.09=13.47	1.08	1.11	47.38	m		2	6.35+6.90=13.25	1.14	1.09	47.92	m
	3	4.55+8.79=13.34	1.07	1.93	34.09	m		3	4.54+8.26=12.80	1.02	1.82	35.45	sm
	4	5.41+6.17=11.58	0.93	1.14	46.73	m		4	5.29+6.05=11.34	0.92	1.14	46.65	m
	5	4.17+7.29=11.45	0.92	1.75	36.38	m		5	4.22+6.78=11.00	086	1.61	38.39	m
	6	4.98+5.68=10.66	0.85	1.14	46.71	m		6	4.83+5.47=10.30	0.83	1.13	46.93	m
	7	4.29+6.23=10.52	0.84	1.45	40.81	m		7	4.25+5.27=9.53	0.82	1.24	44.65	m
	8	3.89+5.27=9.16	0.73	1.36	42.42	m		8	4.01+5.36=9.37	0.76	1.34	42.80	m
MZ3	1	8.00+12.07=20.06	1.60	1.51	39.86	m	BZ	1	7.95+11.74=19.69	1.58	1.48	40.39	m
	2	6.44+7.16=13.60	1.09	1.11	47.37	m		2	6.35+7.05=13.40	1.07	1.11	47.40	m
	3	4.82+8.78=13.59	1.09	1.82	35.43	sm		3	4.88+8.36=13.24	1.06	1.71	36.83	sm
	4	4.02+7.52=11.54	0.92	1.87	34.84	sm		4	5.15+6.37=11.52	0.92	1.24	44.74	m
	5	5.15+6.20=11.35	0.91	1.20	45.37	m		5	4.14+7.15=11.29	0.90	1.73	36.68	sm
	6	4.73+5.73=10.45	0.84	1.21	45.22	m		6	5.05+5.84=10.89	0.87	1.16	46.38	m
	7	4.23+5.63=9.86	0.79	1.33	42.92	m		7	4.41+5.73=10.15	0.81	1.30	43.50	m
	8	4.06+5.49=9.54	0.76	1.35	42.50	m		8	4.23+5.58=9.82	0.79	1.32	43.13	m
NY1	1	8.44+13.25=21.70	1.74	1.57	38.91	m	PD3	1	7.92+11.38=19.30	1.54	1.44	41.03	m
	2	6.53+7.32=13.86	1.11	1.12	47.15	m		2	6.65+7.22=13.87	1.11	1.09	47.93	m
	3	4.32+8.38=12.71	1.02	1.94	34.02	sm		3	4.99+8.46=13.45	1.08	1.70	37.10	sm
	4	4.90+6.23=11.13	0.89	1.27	44.03	m		4	5.56+6.19=11.75	0.94	1.11	47.33	m
	5	3.77+7.34=11.12	0.89	1.94	33.96	sm		5	4.40+7.11=11.51	0.92	1.62	38.23	m
	6	4.95+5.64=10.60	0.85	1.14	46.75	m		6	4.86+5.86=10.73	0.86	1.21	45.35	m
	7	4.23+5.36=9.59	0.77	1.27	44.10	m		7	4.37+5.49=9.86	0.79	1.26	44.28	m
	8	3.82+5.53=9.36	0.75	1.42	40.85	m		8	4.27+5.26=9.52	0.76	1.23	44.81	m

Note: Relative length (%) = 100 × chromosome length / total complement length. Arm ratio = length of the long arm / length of the short arm. m: metacentric; sm: submetacentric.

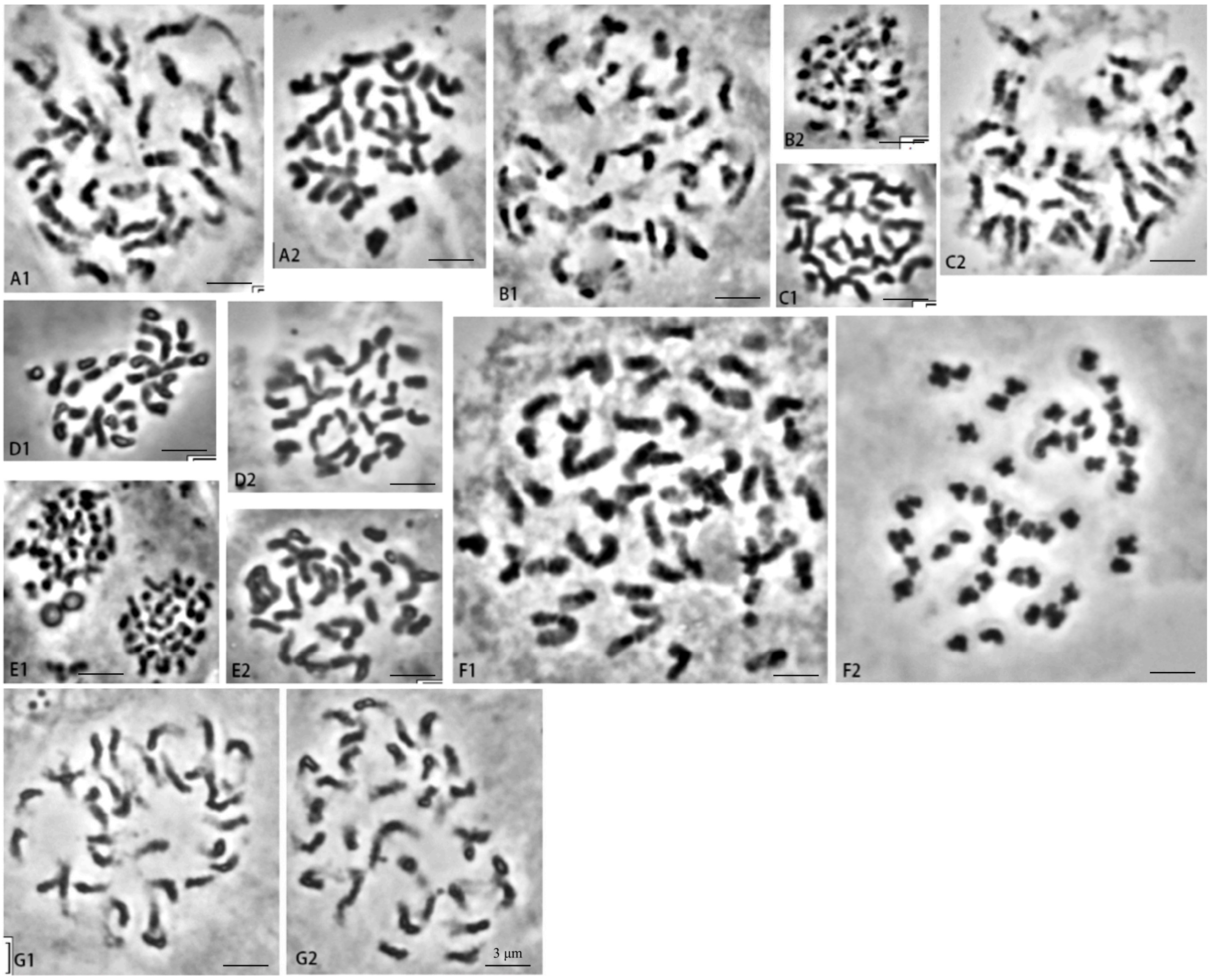

Figure S1. Chromosome number (2*n*) of parental trees in Chinese cherry. **A**: Xichang 1 (4*x* = 32), **B**: Bjjie 7 (4*x* = 32), **C**: Bazhong (4*x* = 32), **D**: Taihe 2 (4*x* = 32), **E**: Nayong 1 (4*x* = 32), **F**: Puding 3 (6*x* = 48), **G**: Luoyang 4 (4*x* = 32). Scale bar represents 3 μm.

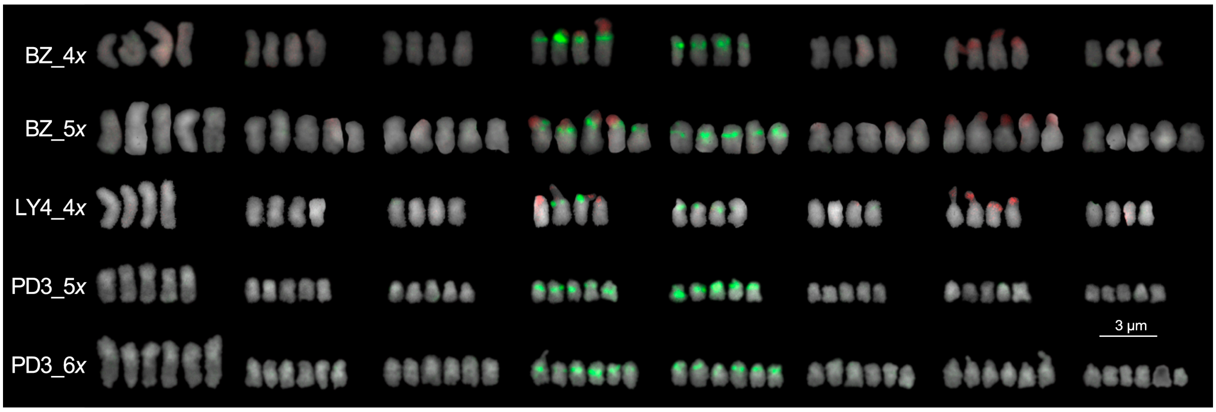

Figure S2. 5S rDNA-FISH on metaphase chromosomes in polyploid Chinese cherry. Green signals indicate 5S rDNA sites. Scale bar represents 3 μm.

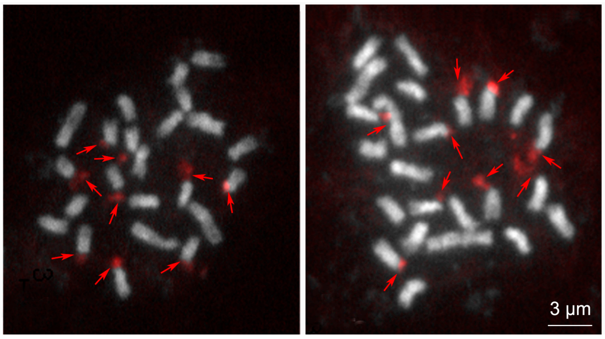

Figure S3. 45rDNA-FISH on metaphase chromosomes in a triploid flowering cherry (Nanjing 2). Red arrows indicate 45S rDNA sites. Scale bar represents 3 μm.
